# Supplementary material for: Rapid Cortical Plasticity Induced by Active Associative Learning of Novel Words in Human Adults
Source: Front Neurosci. 2020 Sep 11;14:895. doi: 10.3389/fnins.2020.00895 (PMC7516206; doi:10.3389/fnins.2020.00895)
Supplement: Supplementary file 1 [file Image_1.PDF]

Source current amplitudes

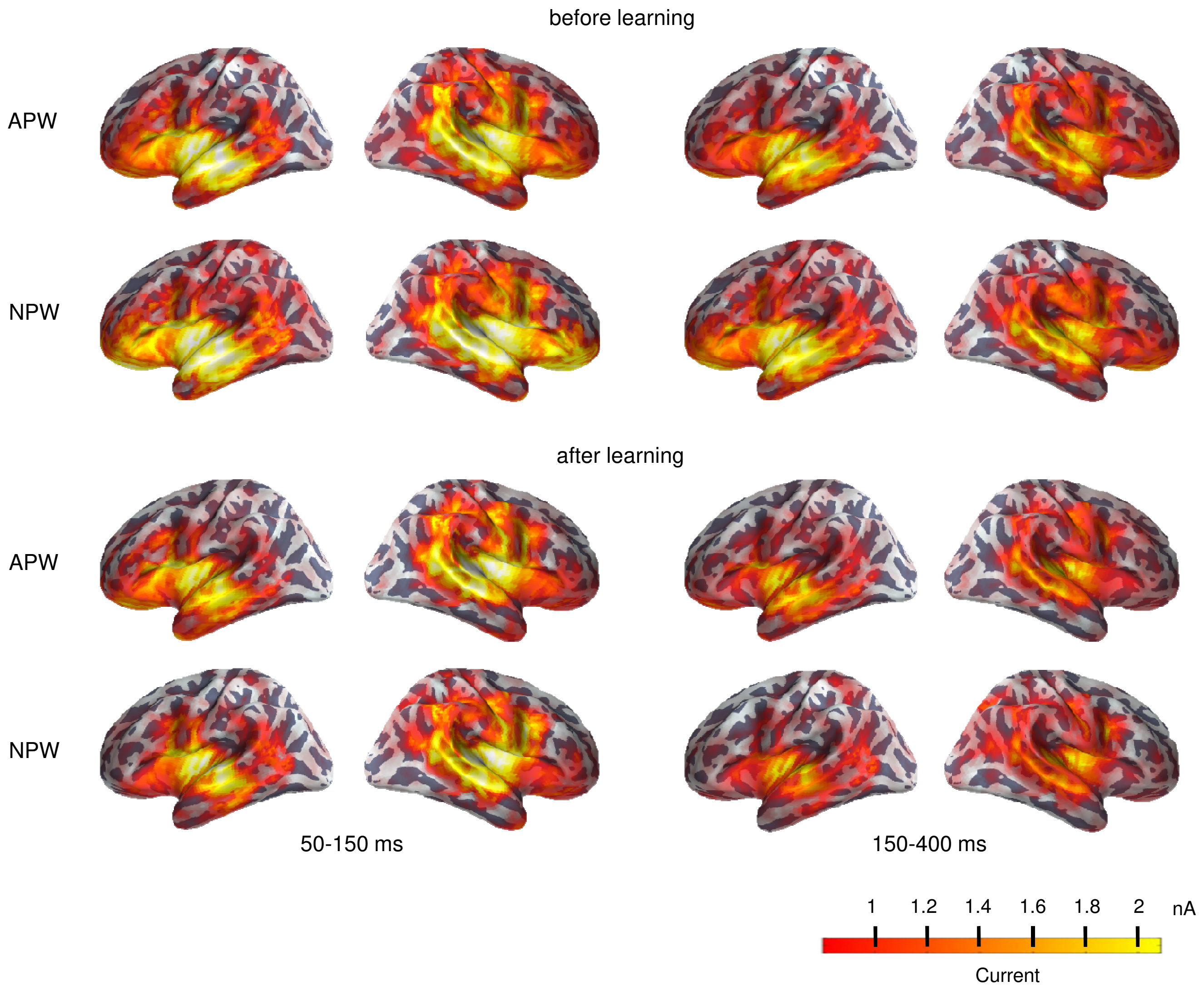

Supplementary figure. Reconstructed cortical sources over the hemispheres for APW and NPW before and after learning.
